# Factors Influencing Bile Duct Injuries: A Dreaded Complication of Laparoscopic Cholecystectomy

**DOI:** 10.7759/cureus.73600

**Published:** 2024-11-13

**Authors:** Anushka Jindal, Nana Yaw Afriyie Badu, Chiko Katiki, V Jaswitha S Ponnapalli, Kesha J Desai, Sadia Mansoor, Lubna Mohammed

**Affiliations:** 1 Surgery, University Hospital Sussex, Worthing, GBR; 2 Surgery, University of Ghana, Accra, GHA; 3 Emergency, American International School of Medicine, Alpharetta, USA; 4 Psychiatry, California Institute of Behavioral Neurosciences & Psychology, Fairfield, USA; 5 Medicine, Medical College Baroda, Vadodara, IND; 6 Internal Medicine, Dow University of Health Sciences, Karachi, PAK; 7 Internal Medicine, California Institute of Behavioral Neurosciences & Psychology, Fairfield, USA

**Keywords:** bile duct injury, cholecystectomy, gall bladder surgery, laparoscopic cholecystectomy complication, post cholecystectomy bile duct injury

## Abstract

Bile duct injuries (BDIs) are dreaded complications of one of the most common general surgical procedures. The injury impacts the quality of life and may have several long-term complications. In some cases, it can also lead to mortality.

This paper aims to review works that have already been published about bile duct injuries and elaborate on the factors leading to it. This includes elaborating on both surgical and non-surgical factors. It also plans to highlight practices and methods to avoid BDIs.

Medical research databases were searched using cholecystectomy and bile duct injuries as keywords. Papers including pre-operative or intraoperative factors, that may cause bile duct injuries, were further shortlisted for this study.

Understanding and knowledge of anatomy plays a key role in bile duct injuries and is essential before performing the surgery. Factors related to the patients, surgeons, and logistics also play a major role in causing bile duct injuries.

Bile duct injuries can be reduced using certain strategies like the B SAFE strategy, R4U line, bail-out methods, imaging techniques along with referrals to Hepatobiliary specialist centers to avoid bile duct injuries.

## Introduction and background

Laparoscopic cholecystectomy (LC) is one of the most common general surgery procedures in today’s time. There has been an 80.4% increase in the number of gallbladder surgeries performed from 2000 to 2019 [1]. There are almost 70,000 cholecystectomies that are done in the UK annually [2]. More than 60,000 of these procedures are performed laparoscopically. LC has replaced open cholecystectomy for most of the uncomplicated and benign indications [3]. This transition is mainly due to minimal scarring and quicker recovery time. LC is now the gold standard for common presentations like symptomatic cholelithiasis, biliary dyskinesia, acalculous cholecystitis, gallstone pancreatitis, gallbladder masses, and gallbladder polyps [4]. Bleeding, infection, conversion to open, port site hernia, and damage to adjacent structures are some of the common complications seen post LC.

Bile duct injuries (BDIs) are the most dreaded complications of cholecystectomies, mainly LC. They have an incidence of 0.3-0.7% [5]. They cause prolonged hospital stays, increase the cost of care, increase the need for invasive procedures, and affect the quality of life [6]. These injuries defeat the purpose of minimally invasive surgery.

Awareness about BDIs has shown a decline in their numbers over the years, although they continue to be the cause of significant morbidity and reduced survival rates. They also come with the risk of litigation [7].

BDIs have been classified by several people. One such classification is the Bismuth classification. This is based on the distance of the injury from the hepatic hilum. Another classification is the Strasberg classification. It includes injuries that are more commonly seen in LC. The Bismuth and Strasberg classification has been discussed in Table 1 below. Other classifications like McMohan classification classify the injuries as major and minor, based on the extent of Common Bile Duct or Cystic Duct- Common Hepatic Duct junction diameter involved. Stewart-Way classification is based on the mechanism of injury (including human factors) and its anatomy [5]. The Stewart-Way classification can be seen in Table 2.

**Table 1:** Bismuth and Strasberg Classification of Bile Duct Injuries RHD: Right Hepatic Duct, CBD: Common Bile Duct, CHD: Common Hepatic Duct

Injury to Bile Duct	Strasberg Classification	Bismuth Classification
Leak from cystic duct or small ducts in the liver	A	-
Aberrant RHD occlusion	B	-
Aberrant RHD leak	C	-
Injury to CBD-Lateral (less than 50% of the circumference involved)	D	-
Stricture in CHD, >2 cm stump	E1	Type 1
Stricture in CHD, <2 cm stump	E2	Type 2
Hilar stricture, Biliary confluence preserved	E3	Type 3
Hilar stricture, confluence involved	E4	Type 4
Stricture to CHD and aberrant RHD	E5	Type 5

**Table 2:** Stewart-Way Classification of Bile Duct Injuries CBD: Common Bile Duct, CHD: Common Hepatic Duct

Injury to Bile Duct	Stewart-Way Classification
CBD mistaken for cystic duct but not divided	Type 1
CHD damaged by clips and cautery	Type 2
CBD mistaken for cystic duct, CBD transected (including at the junction of cystic duct and common bile duct)	Type 3
Right hepatic duct damaged (injury due to dissection or mistaken for cystic duct)	Type 4

Anatomy

Anatomy plays a vital role in surgery. It changes depending on the type and area of the surgery being performed. Cholecystectomies mainly involve the liver, gallbladder and biliary system. Some important terms and anatomical locations in this region include the cystic plate, Calot’s triangle, hepatocytic triangle and bile duct. The segments of the liver (especially segment 4), Rouviere’s sulcus, hepatic artery, umbilical fissure and the enteric structures (duodenum/stomach) are also crucial during the dissection. The above-listed structures help in establishing safety landmarks to correctly identify the anatomy while operating.

Calot’s triangle

Calot’s triangle is an anatomical region that aids in bloodless dissection of the gallbladder and avoiding injuries. It is medially bound by the hepatic duct, laterally by the cystic duct and superiorly by the cystic artery. It is now more commonly referred to as Calot’s region as it often gets distorted in Mirizzi syndrome and malignancies.

Hepatocystic triangle

This is another triangle found adjacent to the gallbladder between the peritoneal layers. It is formed superiorly by the inferior border of the liver, laterally by the cystic duct and medially by the common hepatic duct. The cystic artery, a variable portion of the right hepatic artery, the cystic lymph node, lymphatics, and a variable amount of fibro-fatty connective tissue are found in this triangle. This triangle is used as a landmark to remove the fibro-fatty tissue to achieve Critical View of Safety (CVS). It is also used to locate the cystic artery and facilitate its division while avoiding any injury to the right hepatic artery. It is often affected by the inflammation and may appear thickened, fibrotic, or scarred.

Aberrant anatomy

In a lot of cases, the anatomy around the gallbladder may deviate from what is considered usual. This is termed as aberrant anatomy. In this region, it is usually associated with the cystic duct, the right hepatic artery and at times both the cystic duct and the right hepatic artery. The details of the aberrant anatomy of the cystic duct, the right hepatic artery and both the cystic duct and right hepatic artery are discussed in Tables 3-5, respectively.

**Table 3:** Aberrant Anatomy of Cystic Duct

Type	Anatomy
A	Normal
B	Low insertion into common hepatic duct
C	Parallel cystic and common hepatic duct
D	Absent cystic duct
E	Insertion into right hepatic duct
F	Insertion at biliary confluence

**Table 4:** Aberrant Anatomy of Right Hepatic Duct

Type	Anatomy
A	Normal bifurcation (57%)
B	Trifurcation of 3 ducts (12%)
C1	Right anterior duct draining into common hepatic duct (16%)
C2	Right posterior duct draining into common hepatic duct (4%)
D	Right posterior duct draining into left hepatic duct (5%)
E	Right anterior duct draining into left hepatic duct (1%)
F	Right posterior duct draining into cystic duct (2%)

**Table 5:** Aberrant Anatomy of Right Hepatic Artery and Cystic Artery

Type	Anatomy
A	Cystic artery originating from right hepatic artery or aberrant right hepatic artery
B	Cystic artery originating from left hepatic artery
C	Cystic artery originating from the gastroduodenal artery
D	Cystic artery traveling anterior to common hepatic duct
E	Cystic artery travels anterior to common bile duct and inferior to cystic duct
F	Short cystic artery
G	Multiple cystic arteries

## Review

Methods

This study is a retrospective narrative review. It aims to cover a period of about 24 years spanning from 2000 to the first half of 2024 (January 2024 to June 2024). The review was carried out using a multistep approach. The steps included identifying a research question, relevant keyword, and formation of MeSH keyword. This was followed by shortlisting relevant papers and a detailed analysis of the selected literature.

The research question for this paper was ‘the causes of bile duct injury in laparoscopic cholecystectomies’. The research question aimed at collaborating a surgical intervention ‘Laparoscopic Cholecystectomy’ with an associated complication ‘Bile Duct Injuries’. The keywords used to form the MeSH keywords were: ‘Cholecystectomy’, ‘Laparoscopic Cholecystectomy’, and ‘Bile Duct Injuries’.

The MeSH keywords were: ( "Cholecystectomy/adverse effects"[Majr] OR "Cholecystectomy/complications"[Majr] OR "Cholecystectomy/instrumentation"[Majr] OR "Cholecystectomy/therapeutic use"[Majr] ) AND bile duct injury AND ( "Cholecystectomy, Laparoscopic/adverse effects"[Mesh] OR "Cholecystectomy, Laparoscopic/instrumentation"[Mesh] OR "Cholecystectomy, Laparoscopic/therapeutic use"[Mesh] ). This was formed using the MeSH keyword formation tool on PubMed.

This MeSH keyword was used to find relevant papers. These were further shortlisted by using a systematic approach from electronic databases, including PubMed, Google Scholar, and ScienceDirect.

The inclusion criteria were: papers published during the period starting from 2000 till the first half of 2024 (January 2024 to June 2024) on the topic of BDIs post LCs. The papers included an emphasis on factors causing the BDIs. This included all free full texts available on the above-mentioned databases in the English language.

The population included people belonging to all demographics and geographical locations who underwent the surgical procedure of laparoscopic cholecystectomies and experienced a complication called bile duct injury during the procedure.

All papers outside the time frame of 2000 till the first half of 2024 (January 2024 to June 2024) and not in the English language were excluded. Any papers on bile duct injuries post open cholecystectomies were also excluded from this study.

With the final shortlisted papers, a datasheet was created and the remaining data was analyzed for this study.

Results

BDIs can be caused due to several factors: mainly anatomy, surgeon factors, and patient factors. The most common cause is the inability to identify the anatomy or its misidentification. This accounts for 71-79% of the cases of Bile Duct Injuries. This misidentification or misinterpretation of anatomy is usually caused due to aberrant anatomy. This can include cases of leaks from accessory ducts and gallbladder pathologies like Mirizzi syndrome among others [8]. Clinically it is seen as local inflammation, gallbladder adhesions, and unidentifiable anatomy. Anatomical variation in the form of aberrant anatomy also poses difficulty in delineating structures accurately.

Anatomical factors

New strategies have been developed to aid in establishing anatomical landmarks accurately. These aid in establishing dissection planes and ensure the ligation of correct anatomical structures [7].

The Critical View of Safety (CVS) is a method targeting to correct identification of structures during the procedure. This includes three steps:

1. Clearance of all fatty tissue from the hepatocytic triangle

2. Exposing and ensuring that only two tubular structures are visible entering the gallbladder

3. Separation of the lower third of the gallbladder from the liver to expose the cystic plate

This strategy is designed and used for the correct identification of cystic ducts and arteries although may have limitations in extreme cases [9].

Similarly, B-SAFE is a strategy to allow proper orientation of the planes before starting dissection. It allows the surgeon to identify the anatomical structures that act as boundaries and establish a safe zone for operating. It consists of five structures: B - any visible portion of the bile duct, S - sulcus of Rouviere, A - left hepatic artery, F - umbilical fissure, and E - enteric content - mainly the duodenum.

These structures along with the R4U line allow the surgeon to know when they are entering an area where they may cause a BDI. The R4U line is an imaginary line drawn between Rouviere’s sulcus to the duodenum [10].

Apart from aberrant anatomy, other anomalies like bile draining from other locations or a biliary duct that can be traced till the duodenum could also be variations causing BDIs [11]. Subvesical bile duct is a rare anatomical variation that may cause BDIs if not identified [12]. Gallbladder when contracted or distended due to stones in the neck along with a thickened wall of the gallbladder are some of the other causes [7]. Inflammation (acute or chronic) of the gallbladder can lead to anatomical variation therefore predisposing to increased likelihood of bile duct injuries [5].

Surgeon factors

The experience of the operating surgeon has emerged as a vital factor. Most BDIs are seen in a surgeon's initial LCs. This contributes to the initial learning curve at the beginning of the surgeon's career [13]. Surgeons in training positions or post a fellowship were seen at significantly decreased risk of BDIs than surgeons in non-academic practices. This is suggested to be due to knowledge gaps instead of technical factors [14].

BDIs are predominantly due to the surgeon’s misperception of anatomy. On inspecting the gallbladder and adjacent structures, the surgeon looks for clear uninterrupted boundaries. These are usually inflamed and obscured. In such a situation, the surgeon’s experience and memory come into play and subconscious decisions are made regarding the anatomy. These are acted upon unless there is any visual contradiction [15].

The human factors of surgeons also lead to iatrogenic BDIs. These include a surgeon’s personal concerns, anxiety while operating, and self-doubt. While some surgeons are excessively cautious, others are in a hurry to finish the procedure or overconfident [5]. The stigmata of converting a procedure from laparoscopic to open also play a role [13].

Keeping in mind that every surgeon’s approach and surgical technique may be different, variable techniques like increased traction on the gallbladder can also lead to bile duct injuries. Unsafe use of energy sources like electrocautery or unsafe division of adhesions using sharp instruments may also lead to the burning of the tissue or a partial cut [11,16].

Patient factors

Difficult cases can usually be identified in the patient’s history. These are more prone to BDIs due to their distorted anatomy, unclear surgical field, or need for specialized skills.

The patient’s medical background includes the operative history, physical examination on presentation, lab investigation, and specific imaging. A patient with a prolonged history of presenting symptoms, delayed diagnosis, or delayed presentations is considered at a higher risk. Male patients, age 65 or above, morbid obesity, and high ASA score are some of the key markers of a difficult case. History of upper abdominal surgery, multiple attacks of biliary colic, previous attempts at a cholecystectomy, or liver cirrhosis further add to the risks [11,17].

Raised white cell count above 18000, high CRP and anatomical abnormalities of the gallbladder seen on imaging increase chances of BDIs. Some of the abnormalities seen on imaging may be contracted or shrunken gallbladder, thickened or gangrenous gallbladder, gallbladder perforation, or Mirizzi syndrome. Extrahepatic portal vein thrombosis with portal vein obstruction may further add to the complexity.

This can cause intra-operative inflammation leading to misidentification of anatomy or even inability to identify it [16]. Other intra-operative findings that may also showcase the complexity of the case therefore increasing the chances of bile duct injuries are a small or shrunken gallbladder which is not seen on the first exploration of the area, retracted liver edge near the fundus due to a fissure or puckering or depression [7].

Logistical factors

Geographic distance to and between facilities, availability of correct equipment, level of expertise, and logistics, vary significantly between institutions. These are also factors that may lead to bile duct injuries in an indirect capacity [11].

Delayed referrals, usually more than 72-96 hours [7], due to several factors like delayed admission or delayed diagnosis, or any other factor can also increase the likelihood of bile duct injuries as the cause development of tight adhesions near the neck of the gallbladder due to which it becomes difficult to identify anatomy causing difficulty in dissection [16,18]. Whether the surgery is emergency or elective can also be a cause of bile duct injuries, with the chances of bile duct injuries increasing by threefold in emergency cases [19]. In emergency surgeries, inflammation or excessive bleeding leads to a lack of clarity, difficulty in identifying structures and need for the use of more clips. These lead to a higher risk of bile duct injury [16].

Discussion

Biliary anatomy is complex and subject to variation from patient to patient. Misidentification of the biliary anatomy is seen as the most common reason for bile duct injuries. The main challenge during LC is to correctly identify the anatomy despite the variations [5,20]. Overcoming the anatomical variations is essential to carry out a safe LC. Establishing a CVS before starting to dissect the cystic duct and cystic artery is essential [17]. This is to avoid confusing the cystic duct and artery with the common hepatic duct and hepatic artery [21]. Following the R4U line strictly avoids entry into the dangerous area therefore preventing BDIs.

Chances of biliary injuries in some complex cases, where the cystic structures are non-identifiable, are extremely high. In such cases, a risk assessment of the situation must be done. Time out must to taken to attempt to correctly identify the anatomy. A time-out strategy can be used to minimize bile duct injuries. This means taking time out after entering the abdomen, before the surgeon starts the dissection of the hepatocystic triangle, and also before the cystic duct and cystic artery are clipped and ligated after achieving the CVS [22].

In case the surgeon is unable to proceed despite taking time to evaluate the situation or believes it may be risky, they may bail out. Bailing out may be in the form of abandoning the procedure or conversion to open. In certain cases, a subtotal cholecystectomy in the form of gallbladder fenestration or closure of the neck of the remnants of the gallbladder with a drain in situ may be opted for [23,24]. Surgical techniques like fundus first cholecystectomy have also proven to provide an easier surgical approach [10,17]. Interval cholecystectomy in cases of delayed presentation allows time for the inflammation to reduce, proper imaging, and specialist opinion if needed [25].

Imaging techniques like intraoperative cholangiography (IOC) can also be used during the surgery. IOC is usually used to detect distal common bile duct stones. This technique can also be used to define biliary anatomy during the surgery. It can be helpful in cases where there may be some misunderstanding of the biliary anatomy or inability to see the CVS [26]. Indocyanine green fluorescence cholangiography (ICG-C) is an intravenous infusion of dye that is usually given preoperatively. It is a useful technique that helps in visualizing the structures of the biliary tree, particularly the cystic duct, without the need for X-ray imaging [27]. The technique of using ICG-C is termed near-infrared fluorescence cholangiography (NIRF-C). These imaging techniques are especially recommended in cases of acute cholecystitis [17].

Identifying risk factors in patients based on history and medical background helps in determining and preparing for complex cases. Once identified, such cases may also be referred to more senior or specialized surgeons. They may also be referred to specialized hepatobiliary and pancreatic surgery (HPB) centers for input or even operative management.

Proper work schedules to avoid exhaustion of surgeons along with regular assessment of skills and mental health of surgeons is crucial to avoid complications in all forms of surgery. Proper training in LC with exposure to complex cases under the supervision of senior surgeons can help build confidence and also bridge knowledge and experience gaps.

## Conclusions

Factors causing BDIs can broadly be classified into four major categories, namely anatomical factors, surgeon factors, patient factors, and logistical factors. There are several strategies and techniques to try and avoid bile duct injuries including the ones discussed above. There are emerging concepts like SAGES guidelines for safe cholecystectomy and Culture of Safety in Cholecystectomy (COSIC) that help ensure safer LCs and avoid BDIs.

With proper application of these views, strategies, and methods, along with proper training and exposure to more LCs, BDIs can be reduced further. It is important to understand the impact these injuries have on both, the patient’s life as well as the surgeon who performed the procedure.
